# NBI‐98854, a selective monoamine transport inhibitor for the treatment of tardive dyskinesia: A randomized, double‐blind, placebo‐controlled study

**DOI:** 10.1002/mds.26330

**Published:** 2015-09-08

**Authors:** Christopher F. O'Brien, Roland Jimenez, Robert A. Hauser, Stewart A. Factor, Joshua Burke, Daniel Mandri, Julio C. Castro‐Gayol

**Affiliations:** ^1^ Neurocrine BiosciencesSan DiegoCaliforniaUSA; ^2^ University of South FloridaTampaFloridaUSA; ^3^ Emory UniversityAtlantaGeorgiaUSA; ^4^ Biscayne Bay InstituteMiamiFloridaUSA; ^5^ Research in MiamiMiamiFloridaUSA

**Keywords:** tardive dyskinesia, vesicular monoamine transporter (VMAT2), antipsychotic drugs, randomized controlled trial

## Abstract

**Background:**

Tardive dyskinesia is a persistent movement disorder induced by chronic neuroleptic exposure. NBI‐98854 is a novel, highly selective, vesicular monoamine transporter 2 inhibitor. We present results of a randomized, 6‐week, double‐blind, placebo‐controlled, dose‐titration study evaluating the safety, tolerability, and efficacy of NBI‐98854 for the treatment of tardive dyskinesia.

**Methods:**

Male and female adult subjects with moderate or severe tardive dyskinesia were included. NBI‐98854 or placebo was given once per day starting at 25 mg and then escalated by 25 mg to a maximum of 75 mg based on dyskinesia and tolerability assessment. The primary efficacy endpoint was the change in Abnormal Involuntary Movement Scale from baseline at week 6 scored by blinded, central video raters. The secondary endpoint was the Clinical Global Impression of Change—Tardive Dyskinesia score assessed by the blinded investigator.

**Results:**

Two hundred five potential subjects were screened, and 102 were randomized; 76% of NBI‐98854 subjects and 80% of placebo subjects reached the maximum allowed dose. Abnormal Involuntary Movement Scale scores for NBI‐98854 compared with placebo were significantly reduced (*p* = 0.0005). Active drug was also superior on the Clinical Global Impression of Change—Tardive Dyskinesia (*p* < 0.0001). Treatment‐emergent adverse event rates were 49% in the NBI‐98854 and 33% in the placebo subjects. The most common adverse events (active vs. placebo) were fatigue and headache (9.8% vs. 4.1%) and constipation and urinary tract infection (3.9% vs. 6.1%). No clinically relevant changes in safety assessments were noted.

**Conclusion:**

NBI‐98854 significantly improved tardive dyskinesia and was well tolerated in patients. These results support the phase 3 clinical trials of NBI‐98854 now underway. © 2015 The Authors. *Movement* Disorders published by Wiley Periodicals, Inc. on behalf of International Parkinson and Movement Disorder Society.

Tardive dyskinesia (TD) is a persistent movement disorder that occurs after chronic neuroleptic exposure. Although early studies suggested that the incidence of TD associated with atypical antipsychotic medications was lower than that reported with high‐dose first‐generation antipsychotics, more recent studies suggest that the actual incidence of may be comparable.^1^, ^2^ Classical TD is characterized most commonly by involuntary movements of the orofacial region and choreoathetoid movements in the extremities and trunk, but other forms also exist.^3^ Although at times TD may be of mild intensity, moderate to severe TD can be disabling, significantly impact day‐to‐day functioning and quality of life, and occasionally is life threatening.^4^ The standard tool for assessment of dyskinesia is the Abnormal Involuntary Movement Scale (AIMS), first developed in the 1970s.^5^

No definitive treatment algorithm or approved drug for TD exists in the United States. Although some proposed treatments are based on anecdotal evidence or small blinded trials (e.g., levetiracetam),^6^ others have failed to demonstrate efficacy in larger controlled trials (e.g., vitamin E, amantadine).^7^, ^8^ The first step in treatment is generally to stop or minimize the use of the neuroleptic drug suspected of causing the condition^9^; however, this is not always possible because it may result in an acute exacerbation of TD symptoms and an increased risk for harmful psychiatric decompensation.^10^ Tetrabenazine (TBZ), a vesicular monoamine transporter 2 (VMAT2) inhibitor, is approved for use in TD patients in some countries and has been used “off label” in the United States.^3^

NBI‐98854 is a novel, highly selective, VMAT2 inhibitor in clinical development for the treatment of TD. It has the potential for favorable safety and tolerability and once‐daily dosing. It is an orally active compound with two active metabolites: NBI‐98782 ([+]α‐dihydrotetrabenazine) and its oxidative metabolite NBI‐136110. All three of these entities have highly selective VMAT2 binding, a pharmacological target for hyperkinetic movement disorders.^11^ NBI‐98854 was designed to deliver the active metabolites in a controlled fashion with reduced peak plasma concentrations (ie, low peak‐to‐trough ratio) and low pharmacokinetic (PK) inter‐ and intra‐subject variability. It was also designed to limit off‐target receptor binding (e.g., no affinity for the dopamine D2 receptor) and allow for a favorable safety profile. Clinical pharmacokinetic data indicate that when administered orally, NBI‐98854 is rapidly absorbed, and the active metabolites are formed gradually, with maximum plasma concentration being reached 4 to 10 h after dosing (data on file). The compounds exhibit a half‐life of approximately 20 hours, thereby allowing once‐daily administration.

We report the results of the NBI‐98854‐1202 trial (clinicaltrials.gov listing NCT01733121), conducted to assess efficacy, tolerability, and safety of NBI‐98854 for the treatment of moderate to severe TD.

## Methods

### Study Design

This was a phase 2, randomized, 6‐week, double‐blind, parallel‐group, placebo‐controlled, multi‐center, dose‐titration study to evaluate the safety, tolerability, and efficacy of NBI‐98854 administered once daily.

### Inclusion/Exclusion Criteria

Medically stable male and female subjects, aged 18 to 85 y, with a clinical diagnosis of schizophrenia, schizoaffective disorder, or mood disorder with neuroleptic‐induced TD (as defined by the Diagnostic and Statistical Manual IV),^12^ or gastrointestinal disorder with metoclopramide‐induced TD, were included. Subjects were confirmed to have moderate to severe dyskinesia by a central review of the screening AIMS video. Subjects were required to be psychiatrically stable as determined by the investigator and a Brief Psychiatric Rating Scale^13^ score of less than 50 at screening. Subjects were required to remain on stable doses of their concomitant medications, including dopamine antagonists, for at least 30 d before baseline and during the study. Tetrabenazine and as‐needed use of benzodiazepines, amantadine, or anticholinergic medications were prohibited.

Only subjects deemed to have the capacity to provide consent (using the University of California, San Diego Brief Assessment of Capacity to Consent)^14^ were allowed to sign the informed consent form and participate in the study. The study was approved by institutional review boards of the participating sites.

### Interventions and Drug Administration

Subjects were randomized by a central Interactive Web Response System (IWRS) using computer‐generated block (n = 4 per block) randomization. After screening, subjects returned for baseline assessments (day –1), and eligible subjects were randomized to receive kits containing either NBI‐98854 or matching placebo. On day 1, subjects self‐administered study drug. The once‐daily starting dose of NBI‐98854 was 25 mg, which could be escalated in increments of 25 mg every 2 weeks to a maximum of 75 mg (see Supplemental Data Figure for schematic). The blinded physician investigator (PI) could escalate the subject's daily dose to the next dose level based on clinical judgment of tolerability and persistent dyskinesia, continue with the subject's current dose, or decrease to the subject's prior tolerated dose. Subjects who were unable to tolerate the starting dose or resume the previous dose were discontinued from the study. This process of dose adjustment was applied in a blinded fashion for all subjects (i.e., active and placebo), using otherwise identical study medication kits assigned by a central interactive web response system. Subjects returned for assessments at the end of weeks 2, 4, and 6 (before a decision about potential dose adjustment). A final visit was conducted 2 weeks after the last dose of study drug (end of week 8).

### Endpoints

Severity of TD was assessed by using the AIMS total score (sum of items 1 through 7). The primary efficacy endpoint was the AIMS change from baseline at week 6 as scored by two blinded, central AIMS video raters who were movement disorder specialists otherwise not involved with the study. The scoring process assured that the central raters were blinded to both the treatment assignment and to visit type (e.g., baseline and week 6 examinations presented in random order). Consensus scores of 0 to 4 (none, minimal, mild, moderate, or severe) were reached by the raters for each of the seven body regions in the AIMS after viewing the random‐sequence videos.

The key secondary efficacy endpoint was the seven‐point Clinical Global Impression of Change‐TD scale (CGI‐TD; 1 = very much improved, 7 = very much worse) assessed by the blinded PI who rated global change in TD since baseline. The similar seven‐point Patient Global Impression of Change (PGIC) scale was scored by each subject based on their perception of change in TD since baseline.

### Safety Assessments

Standard clinical chemistry, hematology, electrocardiogram, and urine for drugs of abuse assessments were completed at each study visit. Specific safety scales were assessed at each visit by the PI, including the Barnes Akathisia Rating Scale,^15^ Simpson Angus Scale,^16^ Calgary Depression Scale for Schizophrenia (CDSS),^17^ Positive and Negative Syndrome Scale (PANSS),^18^ Young Mania Rating Scale,^19^ Columbia Suicide Severity Rating Scale,^20^ and Montgomery‐Asberg Depression Rating Scale.^21^

### Statistical Methods

The primary analysis of the AIMS change from baseline was an analysis of covariance of the week 6 scores that included the baseline value as a covariate and treatment and disease category as fixed effects. The modified Intent‐To‐Treat (mITT) and Per Protocol (PP) sets were prespecified for analysis. The mITT set included all subjects randomized who had at least one post‐randomization AIMS for scoring. The PP set excluded any subject who had been randomized to NBI‐98854 but had no measurable drug concentration during the study. Responder analyses were also performed using a prespecified 50% or greater AIMS reduction from baseline and cumulative proportion of responder plots generated.

The sample size calculation for this study was based on data from completed small phase 2 trials. Power was calculated using a two‐sample *t* test with a two‐sided type I error of 0.05 for the NBI‐98854 versus placebo comparison, a standard deviation of 3.1, and an expected treatment mean difference of 3.0. The sample size of 45 subjects per treatment group provided power greater than 0.99.

The CGI‐TD and the PGIC were analyzed using an analysis of variance model and responder analyses. A responder was defined as a subject receiving a score of 1 (“very much improved”) or 2 (“much improved”).

## Results

**Figure 1:**
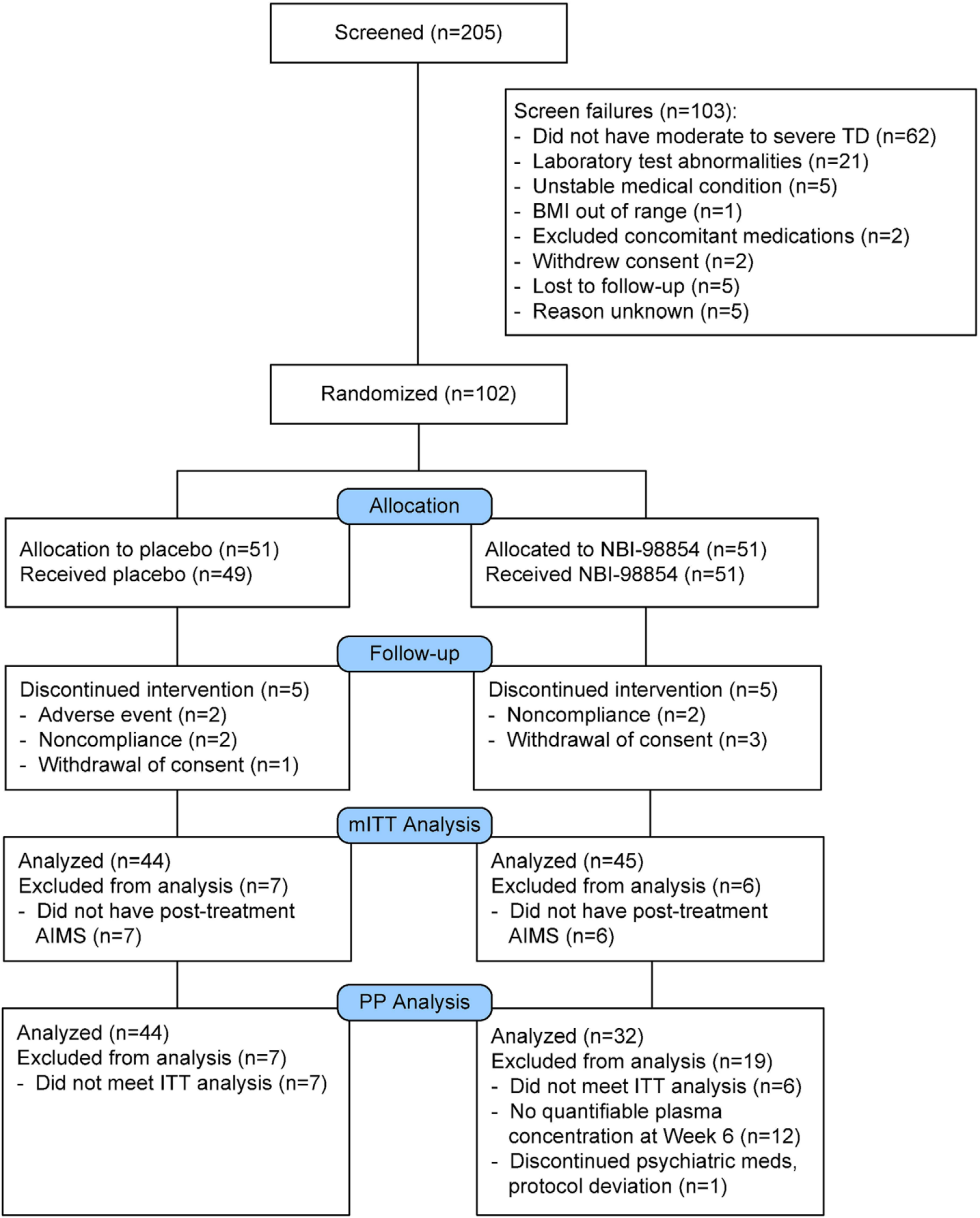
Study Conduct and Subject Disposition [Color figure can be viewed in the online issue, which is available at wileyonlinelibrary.com.]

A total of 205 potential subjects were screened, and 102 were randomized. The safety analysis set included 100 of the 102 randomized subjects (98.0%); two subjects in the placebo group did not receive at least one dose of study drug and were therefore excluded. A total of 13 subjects (12.7%) were excluded from the mITT analysis set (six subjects in the NBI‐98854 group and seven subjects in the placebo group, all because of lack of post‐randomization AIMS), and an additional 11 subjects were excluded from the exploratory PP analysis set (see study drug exposure, discussed later) as presented in Figure 1. The baseline demographic characteristics of study participants are summarized in Table 1; the baseline demographics were comparable, indicating appropriate randomization.

**Table 1:** Demographics and baseline characteristics

Variable	Statistic or Category	Placebo (n = 49)	NBI‐98854 (n = 51)	Total (n = 100)
Age (y)	Mean (SD)	55.6 (9.8)	56.7 (10.8)	56.2 (10.3)
Age at TD diagnosis (y)	Mean (SD)	49.5 (12.1)	48.9 (13.0)	49.2 (12.5)
Gender (n [%])	Male	27 (55.1)	30 (58.8)	57 (57.0)
	Female	22 (44.9)	21 (41.2)	43 (43.0)
AIMS at baseline	Mean (SD)	7.9 (4.5)	8.0 (3.5)	8.0 (4.0)
Disease category (n [%])	Schizophrenia or schizoaffective disorder	30 (61.2)	28 (54.9)	58 (58.0)
	Mood disorder	18 (36.7)	20 (39.2)	38 (38.0)
	Gastrointestinal disorder	1 (2.0)	3 (5.9)	4 (4.0)

TD, tardive dyskinesia; AIMS, Abnormal Involuntary Movement Scale; SD, standard deviation.

### Concomitant Medications

Antipsychotics (including typical and atypical types), antidepressants, and anxiolytics were the most common concomitant medications; drugs in these categories were taken by 40% or more of subjects in each treatment group during the study. All subjects were to take stable doses of their medications 30 days before and during the study; two subjects had a change in antipsychotic dose during the trial, one who was randomized to placebo, the other to NBI‐98854.

### Study Drug Exposure

After the titration protocol, 76% of subjects on active drug reached the 75 mg dose (9 completed on 50 mg, 5 completed on 25 mg), and 80% of placebo subjects reached maximum possible dose. Results are reported for both the mITT and PP populations, the latter excluding any subject with no detectable study drug concentrations (ie, n = 11 of NBI‐98854 subjects, all samples with concentrations below quantitation at all visits).

### Primary Analysis

A statistically significant reduction in the AIMS dyskinesia total score occurred at week 6 from baseline in the NBI‐98854 group compared with the placebo group (mITT, *p* = 0.0005; see Table 2).

**Table 2:** AIMS change from baseline at week 6 (mITT)

	Placebo (n = 44)	NBI‐98854 (n = 45)
Mean (SD)	−1.1 (3.7)	−3.6 (3.5)
Median	−0.5	−3.0
LS mean (SEM)^a^	−0.2 (1.1)	−2.6 (1.2)
95% Confidence interval	(−2.4, 2.0)	(−4.9, –0.3)
LS mean difference (SEM)	−2.4 (0.7)	
95% Confidence interval	(−3.7, −1.1)	
p‐value	0.0005	

a Least‐squares (LS) mean (standard error of the mean [SEM]) based on the analysis of covariance (ANCOVA) model using the modified intent‐to‐treat (mITT) set, with baseline AIMS dyskinesia total score value as a covariate and treatment group and disease category as fixed effects.

Abbreviations: AIMS, Abnormal Involuntary Movement Scale; SD, standard deviation.

Results based on the PP analysis set were similar to that of the mITT analysis set, with significant reductions in the NBI‐98854 group compared with placebo (*p*<0.0001). The mean AIMS reductions at week 6 were comparable in both the schizophrenia and mood disorder subsets (data not shown). A baseline observation carried forward analysis was performed post hoc to assess the impact of the subjects without AIMS score at week 6. Comparable evidence of statistical significance was demonstrated (*p* = 0.0002).

A significantly greater percentage of responders (≥50% reduction in AIMS from baseline) were present in the NBI‐98854 group compared with the placebo group at week 6: 48.9% versus 18.2% in the mITT analysis (*p* = 0.002) and 59.4% versus 18.2% in the PP analysis (*p* = 0.0002).

### Other Endpoints

Marked separation of NBI‐98854 from placebo was also seen on CGI‐TD (Table 3). Most (67%) of the subjects in the NBI‐98854 group were “much improved” or “very much improved,” compared with 16% of subjects in the placebo group (*p* < 0.0001; mITT and PP). Comparable responder rates and separation of active from placebo using the CGI‐TD was seen regardless of underlying diagnosis (62% vs. 15% schizophrenia; 74% vs. 19% mood disorder).

**Table 3:** Summary of CGI‐TD and PGIC scores and response rates by treatment group at week 6 (mITT)

Assessment	Statistic	Placebo (n = 44)	NBI‐98854 (n = 45)
**CGI‐TD score**	Mean (SEM)	3.1 (0.1)	2.3 (0.1)
	LS mean (SEM)^a^	3.1 (0.3)	2.2 (0.3)
	LS mean difference (SEM)	−0.8 (0.2)	
	95% CI	(−1.2, –0.5)	
	*P* value^b^	<0.0001	
**CGI‐TD response:** **“Very much improved” or “Much Improved”**	N (%)	7 (15.9)	30 (66.7)
**PGIC score**	Mean (SEM)	2.9 (0.1)	2.2 (0.1)
	LS mean (SEM)^a^	3.3 (0.3)	2.6 (0.3)
	LS mean difference (SEM)	−0.7 (0.2)	
	95% CI	(−1.1, –0.3)	
	*P*‐value^b^	0.0011	
**PGIC response:** **“Very much improved” or “Much improved”**	n (%)	14 (31.8)	26 (57.8)

a Least‐squares mean (standard error of the mean [SEM]) for scores based on the analysis of variance (ANOVA) model.

b Two‐sided *p* value from ANOVA for comparison of treatment group LS means.

Abbreviations: CGI‐TD, Clinical Global Impression of Change–TD scale; PGIC, Patient Global Impression of Change; mITT, modified intent‐to‐treat; CI, confidence interval.

The proportion of responders based on a 50% reduction in AIMS was comparable with the proportion of responders based on “much improved” or “very much improved” from the CGI‐TD as assessed by the PI. The correlation between the AIMS change from baseline and the CGI‐TD score indicated moderate congruence of scales (*r* = 0.4, *p* = 0.0001). The CGI‐TD responders (ie, score of 1 or 2) had a mean 4‐point reduction in AIMS from baseline. The PGIC scores were also improved for the NBI‐98854 treatment group compared with the placebo group at week 6; see Table 3.

### Adverse Effects

Ten subjects discontinued the trial post‐randomization, five each in placebo and NBI‐98854 groups. None of the discontinuations were attributable to treatment‐emergent adverse events (TEAEs). No deaths or serious TEAEs were reported for any subjects randomized to NBI‐98854. Two subjects randomized to placebo experienced four serious TEAEs; one of these subjects died of a serious adverse event of myocardial infarction, the other was discontinued from the study. The overall incidence of TEAEs was higher for subjects in the NBI‐98854 group (49.0%) compared with the placebo group (32.7%). The most commonly reported were fatigue and headache (9.8% each) for the NBI‐98854 group and constipation and urinary tract infection (6.1% each) for the placebo group (Table 4).

**Table 4:** Incidence of treatment‐emergent adverse events experienced by ≥2 subjects

Adverse Event	Placebo (n = 49) n (%)	NBI‐98854 (n = 51) n (%)
Fatigue	2 (4.1%)	5 (9.8%)
Headache	2 (4.1%)	5 (9.8%)
Decreased appetite	0	4 (7.8%)
Nausea	2 (4.1%)	3 (5.9%)
Somnolence	1 (2.0%)	3 (5.9%)
Dry mouth	0	3 (5.9%)
Vomiting	0	3 (5.9%)
Constipation	3 (6.1%)	2 (3.9%)
Urinary tract infection	3 (6.1%)	2 (3.9%)
Sedation	1 (2.0%)	2 (3.9%)
Back pain	0	2 (3.9%)
Dizziness	2 (4.1%)	0

No clinically relevant changes in mean clinical laboratory assessments, electrocardiogram, and vital sign measurements were noted in either treatment group during the treatment and follow‐up periods. Overall, no safety concerns were expressed related to additional measures for suicidal ideation and behavior, depression, drug‐induced akathisia, or parkinsonism. In addition, there were no concerns from the specific safety measures for schizophrenic symptoms (PANSS) and depression (CDSS, Columbia Suicide Severity Rating Scale, Montgomery‐Asberg Depression Rating Scale) (data on file). Mean scores on these various safety scales were generally low at baseline and remained unchanged or slightly improved.

## Conclusions

NBI‐98854 administered at 25 mg to 75 mg once daily for 6 weeks was associated with a marked reduction in TD severity as assessed by both the AIMS and CGI‐TD and supported by the PGIC. The improvement in dyskinesia (LS mean change from baseline of –2.6 points on NBI‐98854 vs. –0.2 points on placebo; mITT) reflects a greater than 30% reduction in AIMS score. The magnitude of effect is clinically meaningful, especially when looking at responders, that is, greater than 50% reduction in AIMS, and the corresponding CGI‐TD scores of 2 or 1, which reflect the categories of “much improved” or “very much improved.”

This trial design included a unique method for the objective assessment of tardive dyskinesia. The use of movement disorder neurologists as central AIMS video raters appears to be appropriate for TD clinical trials, with excellent sensitivity to detect change. When using random‐sequence video scoring, the potential for placebo and sequence effects appears to be minimized.

Subjects with underlying schizophrenia, schizoaffective disorder, depression, and bipolar disorder receiving concomitant psychotropic polypharmacy appeared to tolerate NBI‐98854 well at these doses. No adverse events were seen related to depression or worsening of psychiatric safety scales. The absence of signs of monoamine depletion is notable given the shared mechanism of action with tetrabenazine, a VMAT2 inhibitor administered three times daily and approved in some countries for use in TD patients. No large, placebo‐controlled trials of TBZ for TD have been published. However, safety and tolerability data for TBZ are available from a controlled trial in Huntington's disease.^22^ In that pivotal HD trial, adverse events were common during the TBZ titration phase, with dose reductions required because of sedation, akathisia, parkinsonism, and depression. NBI‐98854, with once daily dosing and the potential for low rates of these particular adverse events, may be a useful alternative therapy. A formal comparison trial with adequate long‐term administration would be the only way to compare safety and tolerability of these medications.

As revealed by the pharmacokinetic analyses, not all subjects randomized to NBI‐98854 were actually compliant with investigational drug as instructed. The absence of any detectable concentrations of NBI‐98854 or metabolites at multiple time points indicates that these subjects may have enrolled for secondary gain such as the stipend for participation or free medical assessments.

Limitations of this trial include modest sample size and 6‐week treatment duration. Because TD is a chronic condition, particularly for patients who require chronic antipsychotic medication, longer‐term studies are warranted for determination of sustained efficacy and safety risks. A larger database of patients exposed to study drug will be necessary for regulatory review and potential approval. This study was neither designed nor powered to allow formal comparison of efficacy by individual doses or underlying diagnoses. It is possible that doses greater than 75 mg might provide additional benefit for some subjects. Finally, the low number of gastrointestinal disorders subjects with metoclopramide‐induced TD enrolled in this trial limits conclusions regarding potential safety and efficacy of NBI‐98854 for such patients.

Tardive dyskinesia is a potentially serious condition with no approved treatment options in the United States. The positive efficacy data for AIMS, CGI, and PGIC in this study are encouraging. NBI‐98854 appears to be well tolerated at the doses studied in patients with moderate or severe TD and underlying psychiatric illness. The positive results and lack of specifically approved TD therapies indicate that larger and longer‐duration phase 3 clinical trials of NBI‐98854 for TD are warranted (and have been initiated), particularly in light of the escalated use of antipsychotic medication for a range of clinical conditions.

## Author Roles

1. Research Project: A. Conception, B. Organization, C. Execution; 2. Statistical Analysis: A. Design, B. Execution, C. Review and Critique; 3. Manuscript Preparation: A. Writing the First Draft, B. Review and Critique.

C.F.O.: 1A, 1B, 2C, 3A

R.J.: 1B, 1C, 3B

R.A.H.: 1A, 1C, 3B

S.A.F.: 1A, 1C, 3B

J.B.: 2A, 2B, 3B

D.M.: 1C, 3B

J.C.‐G.: 1C, 3B

## Financial Disclosures

D.F.M. and R.A.H. have research contracts from Neurocrine Biosciences, Inc.; R.A.H. has served as a consultant for Allergan Neuroscience, Pfizer, UCB Biosciences, Teva, Impax Pharmaceuticals, and Auspex Pharma; on the Steering Committee for Chelsea; and on the Advisory Boards of Auspex Pharma, Lundbeck, AstraZeneca, and Acadia. S.F. has received honoraria from Merz, Chelsea Therapeutics, Neurocrine, Lundbeck, Auspex, Avanir, UCB; and grants from TEVA, Ipsen, Allergan, Medtronics, Auspex, Genzyme Corp, Genzyme A Sanofi, the Michael J Fox Foundation, and the NIH; and has received royalties from Demos, Blackwell Future for textbooks, Uptodate, Neurotherapeutics; and has a research contract with Neurocrine Biosciences, Inc. J.C.C.‐G. has a research contract with Neurocrine Biosciences, Inc. C.F.O.'B., R.J., and J.B. report no financial discosures.

## Supporting information

Additional Supporting Information may be found in the online version of this article at the publisher's web‐site.

Supporting Information
