# Supplementary figures and images for: NBI‐98854, a selective monoamine transport inhibitor for the treatment of tardive dyskinesia: A randomized, double‐blind, placebo‐controlled study
Source: Mov Disord. 2015 Sep 8;30(12):1681–7. doi: 10.1002/mds.26330 (PMC5049616; doi:10.1002/mds.26330)

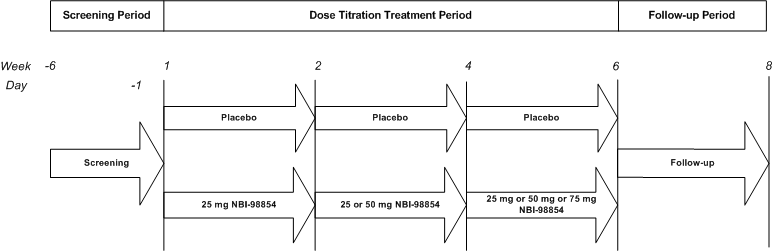

Supplement: Supplementary file 1 — Supporting Information [file MDS-30-1681-s001.tif]
